# Beneficial Effects of Isoflavones in the Kidney of Obese Rats Are Mediated by PPAR-Gamma Expression

**DOI:** 10.3390/nu12061624

**Published:** 2020-06-01

**Authors:** Edson de Andrade Pessoa, Márcia Bastos Convento, Bianca Castino, Ala Moana Leme, Andréia Silva de Oliveira, Alef Aragão, Sheila Marques Fernandes, Adriana Carbonel, Cassiane Dezoti, Maria de Fátima Vattimo, Nestor Schor, Fernanda Teixeira Borges

**Affiliations:** 1 Nephrology Division, Department of Medicine, Universidade Federal de São Paulo, São Paulo SP 04023-900, Brazil; nefro.edson@hotmail.com (E.d.A.P.); convento@unifesp.br (M.B.C.); alamoanah@hotmail.com (A.M.L.); andreiaoliveira9921@gmail.com (A.S.d.O.); tukabc@hotmail.com (N.S.); 2 Interdisciplinary Postgraduate Program in Health Sciences, Universidade Cruzeiro do Sul, São Paulo SP 01506-000, Brazil; bcastino95@gmail.com (B.C.); alef931127@hotmail.com (A.A.); 3 Experimentation Laboratory in Animal Model, School of Nursing, Universidade de São Paulo, São Paulo SP 05403-000, Brazil; sheilamfernandes@usp.br (S.M.F.); cassianedezoti@usp.br (C.D.); 4 Histology and Structural Biology Division, Department of Morphology and Genetics, Universidade Federal de São Paulo, São Paulo SP 04039-032, Brazil; adricarbonellfisio@hotmail.com; 5 Department Medical-Surgical Nursing, School of Nursing, Universidade de São Paulo, São Paulo SP 05403-000, Brazil; nephron@usp.br

**Keywords:** high-fat/high-fructose diet, nephropathy, isoflavones, PPAR-γ, angiotensin II, oxidative stress

## Abstract

Several studies have demonstrated an important association between altered lipid metabolism and the development of kidney injury because of a high-fat diet. Fructose is also closely associated with renal injury. We opted for a combination of fructose and saturated fats in a diet (DH) that is a model known to induce renal damage in order to evaluate whether soy isoflavones could have promising use in the treatment of renal alterations. After two months of ingestion, there was an expansion of visceral fat, which was associated with long-term metabolic disorders, such as sustained hyperglycemia, insulin resistance, polyuria, dyslipidemia, and hypertension. Additionally, we found a decrease in renal blood flow and an increase in renal vascular resistance. Biochemical markers of chronic kidney disease were detected; there was an infiltration of inflammatory cells with an elevated expression of proinflammatory cytokines (tumor necrosis factor-α, interleukin (IL)-6, and IL-1β), the activation of the renin–angiotensin system, and oxidative/nitrosative stress. Notably, in rats exposed to the DH diet for 120 days, the concomitant treatment with isoflavones after 60 days was able to revert metabolic parameters, renal alterations, and oxidative/nitrosative stress. The beneficial effects of isoflavones in the kidney of the obese rats were found to be mediated by expression of peroxisome proliferator-activated receptor gamma (PPAR-γ).

## 1. Introduction

The Occidental diet (OD) and lifestyle have led to the pandemic of obesity [1,2,3,4]. The OD is an important risk factor for kidney function and the development of chronic kidney disease (CKD) [5,6,7]. Some of the damaging renal consequences of obesity may be mediated by downstream comorbid conditions such as hypertension or diabetes mellitus (DM). Additionally, there are effects of adiposity that could directly impact the kidneys and that are induced by the endocrine activity of the adipose tissue [8]. These include the development of inflammation, insulin resistance, oxidative stress, abnormal lipid metabolism, and the activation of the renin–angiotensin system (RAS) [9]. 

The OD is characterized by an over availability of food (with high-sugar desserts and drinks), high intakes of high-fat foods, and high intakes of animal fat with high levels of saturated fats and trans-fatty acids, high-fat dairy products, red meat, and refined grains. Indeed, corn fructose is highly consumed in the OD (instead of glucose in desserts, condiments, and carbonated beverages) as a sweetening substitute [10,11].

The adverse effects of dietary saturated fats and fructose on kidney function have been supported by numerous studies [12,13,14,15,16,17,18,19,20,21]. However, no study has evaluated the effect of the combination of fructose and high-fat diet in the kidney function. Concerning fats, the excessive renal fat deposition can lead to tubular cell injury [16], tubulointerstitial fibrosis [17], structural glomeruli alterations [18], mesangial sclerosis, and podocyte damage [19]. Fructose is also closely associated with renal injury [12,13,14,15], as its ingestion can accelerate established renal diseases, with greater degrees of proteinuria, focal tubulointerstitial injury, and glomerulosclerosis.

More studies are necessary to establish renal effects of high sugar, salt, fat, and protein dietary contents to uncover the participation of each component for kidney function. However, in this study, we opted for a combination of fructose and saturated-fats in a diet to induce renal damage [12,13,14,15,16,17,18,19,20,21] and to evaluate whether soy isoflavones could have promising use in the treatment of renal alterations [22].

The isoflavones are phenolic chemical compounds that belong to the class of phytoestrogens; they are widely distributed in the plant kingdom. The main isoflavones found in soy protein and their derivatives are genistein, glycitein, and daidzein [22,23]. 

Over two decades, vigorous research studies have been performed on the effects of isoflavones as an alternative treatment for menopausal symptoms for people who cannot or are unwilling to take hormone replacement therapy (HRT) for a variety of reasons concerning the fear of cancer and other adverse effects. Moreover, in literature, there is still no consensus on the effects of isoflavones on HRT. Isoflavones are thought to be responsible for exerting estrogen-like effects, especially by affinity of isoflavones to estrogen receptors-β (ERβ) [24]. Daidzein can further convert to S-equol, a compound structurally similar to estrogen, intestinally, and it also preferentially binds to ERβ and has a higher transcriptional expression than isoflavones. However, the efficacy of the alleviation of hot flashes in postmenopausal and perimenopausal women was shown in 19 potentially relevant publications [25].

Isoflavones have also been the most extensively studied protein in patients with CKD and have demonstrated renal protective properties in several clinical studies [23,26,27,28,29]. However, these studies in humans have been limited to a clear understanding of the molecular and cellular role of isoflavones in maintaining renal function.

Isoflavones have been found to be beneficial in a wide range of diseases and have beneficial effects on some physiological risk factors for chronic kidney disease, such as dyslipidemia [30], hypertension [31], DM [32], and obesity [33]. In vitro studies [34,35,36] have shown that isoflavones downregulate several proinflammatory mediators like tumor necrosis factor-alpha (TNF-α), interleukin (IL)-6, IL-8, IL-1β, prostaglandin E2 (PGE2), monocyte chemoattractant protein-1, and intercellular adhesion molecule-1, or they upregulate anti-inflammatory cytokines like IL-10 [37]. Other studies have shown that isoflavones can exert antioxidant effects in reactive nitrogen species (RNS), reactive chlorine species (RCS), and reactive oxygen species (ROS), which—when imbalanced—cause tissue injury that leads to the development of chronic or acute inflammation in various diseases [37].

The nuclear transcription factors peroxisome proliferator-activated receptor gamma (PPAR-γ) and PPAR-α, among different potential candidates, could be essential targets for mediating the isoflavones’ effects [37,38,39]. 

PPARs are nuclear hormone receptors that act as transcription factors upon ligand binding. PPAR-α regulates catabolism, mainly in the liver and the heart, and PPAR-γ1 is highly expressed in adipose tissue but is also expressed in kidney cells. It modulates physiological processes such as lipid and glucose metabolism by acting in several organs, including the kidney [39,40,41].

PPAR-γ controls the expression of networks of genes involved in maintaining metabolic homeostasis through the direct regulation of genes involved in glucose metabolism such as glucose transporter 4 (GLUT4), phosphoinositide 3 kinase (PI3K), insulin receptor substrate 1 (IRS-1), insulin receptor substrate 2 (IRS-2), and catabolite activator protein (CAP), as well as genes involved in lipid metabolism such as fatty acid-binding protein (aP2), acyl-CoA-binding protein (ACBP), lipoprotein lipase (LPL), cluster of differentiation 36 (CD36), phosphoenolpyruvate carboxykinase (PEPCK), acyl-CoA synthetase (ACS), and glycerol kinase (GyK) [42].

PPAR-γ1 is expressed in podocytes, mesangial, and endothelial cells [43], and it may have an essential role in kidney function. The beta-oxidation of free fatty acids is the main source of substrates for ATP synthesis in proximal tubules [44]. Interestingly, a mutation in enzymes involved in lipid metabolism decreases glomerular filtration, corroborating the interaction between lipid metabolism and kidney function. Additionally, lipid-mediated kidney damage is lower in PPAR-γ heterozygous knockout mice [21].

The kidney contributes to glucose metabolism through gluconeogenesis, corresponding to 20% of glucose production during prolonged fasting and also through the proximal tubular reabsorption of glucose [44]. PPAR-γ agonists inhibit gluconeogenesis in a proximal tubule, but, more importantly, they have protective effects in diabetic nephropathy by preventing albuminuria [45]. Furthermore, PPAR-γ and PPAR-α are found in inflammatory and immune cells such as T and B cells, dendritic cells, monocytes, macrophages, and vascular cells, thus linking them to a role in inflammatory responses [37,46,47]. Indeed, synthetic PPAR-γ agonists, including pioglitazone, troglitazone, and rosiglitazone, were shown to suppress the production of cytokines by inflammatory cells [47].

It is known that isoflavones (genistein, daidzein, and glycitein) can activate both PPAR-α and PPAR-γ—studies have shown that several structurally distinct isoflavones can activate PPARs with similar efficacies [42]. In support of this hypothesis, Matin et al. [43] published a comprehensive structure-activity study demonstrating that 7-hydroxy-benzopyran-4-one structure that comprises the core structure of isoflavones is the key for PPAR-γ and PPAR-α activation. The 7-hydroxy-benzopyran-4-one structure was designed via a proposed cyclization of 1,3-diaryl-2-propenones, and it exhibits a high resemblance to the core structure of both thiazolidinediones (TZDs) and fibrates.

Several in vitro studies demonstrated that isoflavones can induce the expression of PPARs in endothelial cells, monocytes/macrophages, liver cells, and bone marrow stromal cells under pathological conditions [37,38,39]. Also, in vivo studies [37,48,49,50,51,52,53] have documented that isoflavones can activate both PPAR-γ and PPAR-α to prevent metabolic syndrome and DM through mechanisms ranging from the improvement of lipid homeostasis to insulin sensitivity. Nevertheless, no studies have evaluated the effects of isoflavones on PPAR-γ signaling in kidney tissue in obesity animal model.

Thus, the goal of this study was to evaluate the therapeutic effect of isoflavones on the kidneys of rats, where obesity was induced by a high-fat/high-fructose diet and discriminated the influence of PPAR-gamma in this environment.

## 2. Methods

### 2.1. Animals, Experimental Design, and Diets

**Figure 1:**
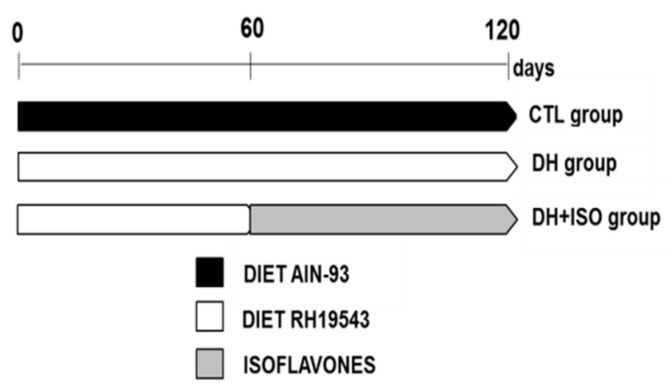
Experimental design and composition of the diets. The diets for the respective groups were as follows: the control diet group (CTL) had 20.56% protein, 61.74% carbohydrates, 17.70% lipids, 10% sucrose (glucose and fructose), and 3.9 kcal/g (AIN-93, Rhoster Indústria e Comércio Ltda., SP, Brazil); the high-fat/high-fructose diet group (DH) had 15.32% protein, 16.41% carbohydrates, 68.27% lipids, 20% fructose, 35% lard, and 5.6 kcal/g (RH19543, Rhoster Indústria e Comércio Ltda., SP, Brazil); and the high-fat/high-fructose diet and isoflavones group (DH and ISO) had isoflavones (Ultra Soy Extract, Life Extension, FL, USA) concomitantly administered by gavage with the DH diet during the last 60 experimental days The treatment of animals with ISO was standardized at 300 mg/kg and diluted in propylene glycol, where 45% was isoflavones containing genistein (51.85%), daidzein (40.74%), and glycitein (7.41%).

The experimental protocol in vivo was approved by the ethics committee (Research Ethics Committee 2014/0054) of the Universidade Federal de São Paulo and was performed in accordance with the Brazilian guidelines for scientific animal care and use [54,55]. Male Wistar rats weighing between 190 and 210 g at 6 weeks of age were randomly assigned to the control group (CTL), the high-fat/high-fructose diet group (DH), and the high-fat/high-fructose diet and isoflavones group (DH and ISO) (Figure 1). The animals were subjected to the experimental protocol for a total of 4 months, which was repeated 3 times at different moments with N = 5 for each group. Chow and water were supplied ad libitum. Food consumption and body weight were monitored weekly to carefully characterize weight gain and calorie intake.

The rats were kept under controlled environmental conditions (12/12 h light/dark cycles, and 22–24 °C), in individual boxes with wood shavings. At baseline, 30, 60, 90, and 120 days after the initiation of the experimental protocol, the animals were placed in metabolic cages for 24 h for urine collection, and blood samples were collected from the lateral tail vein. The rats were euthanized 120 days after the beginning of the experimental protocol through the intraperitoneal injection of a toxic dose of xylazine (10 mg/kg)/ketamine (90 mg/kg; Agribands do Brasil Ltda., SP, Brazil), and both kidneys were then removed for immunohistochemistry and Western blotting. 

### 2.2. Measurement of Visceral Fat 

The measurement of visceral fat comprised measuring the mesenteric, retroperitoneal, and epididymal fats. An AD-5000 balance (Marte Cientfica Ltda., SP, Brazil) was used, and the measurement of visceral fat was calculated as the ratio of total adipose tissue weight to body weight × 100. The results are expressed in grams.

### 2.3. Measurement of Systolic Blood Pressure (SBP)

The systolic blood pressure (SBP) was indirectly measured using tail plethysmography. The animals were placed in a warm chamber for 10 min, and cuff and wrist receivers were attached to the tail. SBP was recorded using an electric sphygmomanometer coupled to a 2-channel Gould model 2200 S polygraph (Record 2200S, Gould Inc., OH, USA), and the results are expressed as mean ± standard error.

### 2.4. Biochemical Analysis

The plasma and/or urine levels of creatinine, urea, uric acid, total cholesterol, high-density lipoprotein (HDL), and triglycerides were spectrophotometrically assayed according to standard procedures using commercially available diagnostic kits (Labtest Diagnostic, MG, Brazil). The levels of creatinine were determined with a colorimetric method based on the Jaffé reaction [56]. The levels of urea were determined with a colorimetric assay based on urease activity [57]. HDL, total cholesterol, and uric acid levels were determined with a colorimetric assay based on antipyrilquinonimine, whereas triglyceride determination was based on quinonimine. The results are expressed as mg/dL.

Creatinine clearance was calculated according to the equation: (urine creatinine concentration × urine volume)/(plasma creatinine concentration × 1440). The results are expressed as mL/min.

The urine sodium concentration (FE_Na_) was determined with a Micronal B462 flame photometer (Micronal, SP, Brazil), and was calculated according to the equation: (urinary sodium concentration × plasma creatinine concentration)/(plasma sodium concentration × urinary creatinine concentration) × 100. The results are expressed as the percentages. 

The urinary proteins were determined with a colorimetric method based on pyrogallol red-molybdate [58]. The results are expressed as mg/24 h.

The plasma levels of glucose were determined with commercially available diagnostic kits (Accu-Chek Boehringer Mannheim, IN, USA). The glucose levels are expressed as mg/dL.

The plasma levels of glycated hemoglobin were determined with commercially available diagnostic kits (Alamar Tecno Científica Ltda., SP, Brazil). The results are expressed as percentages.

The insulin tolerance test (ITT) is a simple and convenient in vivo method for evaluating insulin action. The plasma glucose disappearance rate (kITT) was calculated from the linear slope of the plasma glucose concentration curve between 0, 10, 20, and 30 min, as described previously [59]. The results are expressed as percentages/min. 

### 2.5. Systemic Hemodynamics

The heart rate (per minute/bpm), renal vascular resistance (RVR), and renal blood flow (RBF) were determined in the anesthetized rats at 121 days of experimental protocol in all groups. Subcutaneous needle electrodes were introduced into the upper limbs and left lower limbs for electrocardiographic recording in lead II (DII) to obtain a heart rate and the evaluation of the cardiac rhythm pattern. A tracheostomy was performed with polyethylene cannula (PE 240). In the left carotid artery, we conducted a catheterization (PE 50), where we coupled a pressure transducer (SP844, 50 μV/V/cm Hg 1–10 v, MEMSSAP) to monitor mean arterial pressure. 

The abdominal cavity was accessed, and we performed a unilateral nephrectomy on the right kidney. We located the left renal artery, and this was delicately dissected for the coupling of an ultrasonic probe (Transonic Systems Inc., NY, USA) for the evaluation of RBF. 

Renal vascular resistance was calculated using the following equation: RVR = MAP/RBF, where MAP is the mean arterial pressure. The results of RBF and RVR were corrected for 100 g/wt of their respective animals and are expressed as the average. The results are presented as mean ± standard error.

### 2.6. Immunohistochemistry

Paraffin sections were subjected to alcohol and xylene gradient solutions, antigen retrieval, protein block, and incubation with primary antibodies against CD31 (1:200, mouse clone JC70a endothelial, M082, Dako, CA, USA), CD68 (1:200, mouse monoclonal clone, M0876, Dako, CA, USA), and kidney injury molecule 1 (KIM-1) (1:200, rabbit IgG, H07H, Sino Biologica, BJ, CH) overnight at 4 °C. After this time, the sections were incubated with streptavidin-peroxidase for 30 min (Dako, CA, USA). 

The obtained microscope images were calculated using the Leica DFC 310 FX image analysis software (Leica do Brasil Importação e Comércio Ltda., SP, Brazil) and are expressed as a percentages/stained area.

### 2.7. Western Blotting

The protein concentration was verified by the method of Lowry [60]. The kidney tissues were lysed with a 200 μL of a RIPA lysis buffer and centrifuged at 12.000× *g* for 5′ at 4 °C; 30 μg of proteins were separated by 10% polyacrylamide gel electrophoresis and transferred to polyvinylidene fluoride membranes using a Mini Trans-Blot Electrophoretic Transfer Cell (Bio-Rad, CA, USA). The nonspecific binding sites were blocked with 5% albumin (*v*/*v*) in tris-buffered saline. The blots were incubated overnight at 4 °C with renin (1:500, Santa Cruz Biotechnology, TX, USA), angiotensinogen (1:500, Santa Cruz Biotechnology, TX, USA), angiotensin I (1:500, Santa Cruz Biotechnology, TX, USA), angiotensin II (1:500, Santa Cruz Biotechnology, TX, USA), IL-6 (1:500, Santa Cruz Biotechnology, TX, USA), IL-1β (1:500, Santa Cruz Biotechnology, TX, USA), TNF-α (1:500, Santa Cruz Biotechnology, TX, USA), PPAR-γ1 (1:500, Abcam, MA, USA), and β-actin (1:500, Abcam, MA, USA) primary antibodies. 

After washing with tris-buffered saline, the membranes were incubated for 1 h at 4 °C in enzyme horseradish peroxidase (HRP)-conjugated secondary antibodies (1:10.000; Cell Signaling, MA, USA). The immunoreactive protein bands were visualized using Pierce ECL Plus Chemiluminescent substrate detecting reagents (Thermo Fisher, MA, USA). Images were obtained with an Alliance 7 Chemiluminescence documentation system (UVItec, Cambridge, UK). The immunoblot band intensities were quantified using ImageJ software and are expressed as a ratio/β-actin. 

### 2.8. Oxidative and Nitrosative Stress 

The malondialdehyde (MDA) combined with thiobarbituric acid (TBA) for the determination of thiobarbituric acid reactive substances (TBARS) in urine, forming a red compound whose concentration was assessed by spectrophotometry with reading at 535 nm [61]. The lipid peroxidation levels are expressed as nmol/mg of urinary creatinine. 

Urinary peroxides were determined by the ferrous oxidation of the xylenol orange version 2 (FOX-2) method [62]. The results are expressed as nmol/mg of urinary creatinine. 

Nitric oxide (NO) was determined in urine samples using the Griess method [63]. The results are expressed as nmol/mg of urinary creatinine.

### 2.9. Statistical Analysis

The descriptive statistical analysis of the data was performed with the Action Stat software for Windows (version 3.3.2) while considering a significance level of 5% (*p* < 0.05). The data were analyzed using the Shapiro–Wilk normality test. The data with a normal distribution were analyzed using the Bonferroni post-hoc test, whereas the data with non-normal distribution were analyzed using the Kruskal–Wallis test. 

## 3. Results 

### 3.1. Metabolic Parameters

The DH group was fed a high-fat/high-fructose diet for 120 days to induce obesity, whereas the DH and ISO group was fed high-fat/high-fructose diet for 120 days; however, after 60 days, this group was concomitantly treated with isoflavones until the end of the experimental protocol. Both groups had a significantly higher consumption of food, calorie ingestion, and progressive weight gain than the CTL group (Table 1). 

Visceral fat was analyzed from three different compartments, namely mesenteric, retroperitoneal, and epididymal fats. The DH and DH group and the ISO group acquired a significantly larger amount of fat in these compartments at the end of the experimental protocol than the CTL group. However, the DH and ISO group presented a lower amount than the DH group. 

There was a significant increase in the triglycerides and total cholesterol, as well as a significant decrease in the HDL cholesterol in the rats fed the DH diet when compared to those fed the control diet. In contrast, the DH and ISO group presented lower levels of triglycerides (90 and 120 days) and total cholesterol (120 days), as well as higher HDL cholesterol levels (120 days) than the DH group (Table 2)

Glycated hemoglobin is a robust biomarker of average glucose levels of the previous two-to-three months [64]. At 120 days, its levels were doubled in the rats fed the DH diet when compared to those in the CTL diet group. In the rats exposed to the DH and ISO diet, we observed a significant decrease when compared to that the DH group (Table 3).

An increase in glucose levels was observed in all experimental groups after 30 days of experimental protocol when compared to the CTL group at the same experimental period, whereas the rats exposed to the DH diet for 120 days and treated concomitantly after 60 days with isoflavones (DH and ISO) presented lower levels than the DH group at 120 days (Table 3).

We observed that the rats in the DH group presented with polyuria after 90 days. However, the rats exposed to the DH diet for 120 days and treated concomitantly with isoflavones after 60 days (DH and ISO) showed an improvement in polyuria at 120 days when compared to the DH group (Table 3).

At the final period of the experiment (120 days), the insulin tolerance test was performed, and an increase in insulin resistance was observed in the DH group (10, 20, and 30 min) when compared to the control group. However, insulin levels in the rats exposed to the DH diet for 120 days and treated concomitantly after 60 days with isoflavones (DH and ISO) were not statistically different when compared to those of the CTL group (Table 3). 

### 3.2. Evaluation of Renal Function

**Figure 2:**
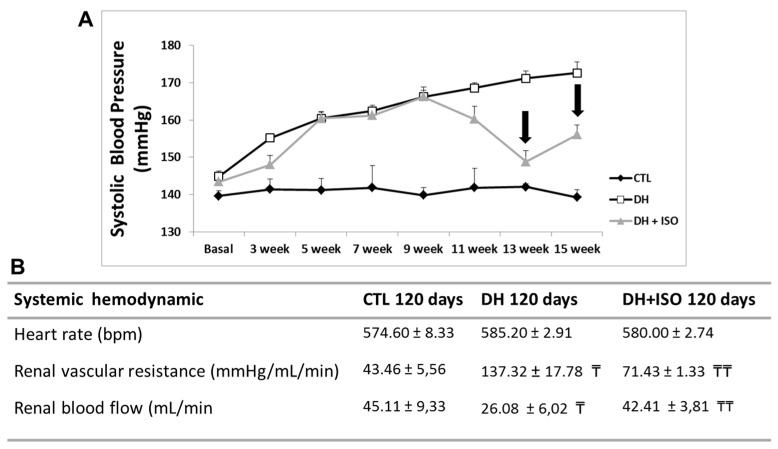
Systolic blood pressure and systemic hemodynamics. Rats in the CTL group, the DH group, and the DH and ISO group. (**A**) Measurement of the systolic blood pressure (SBP) in rats at baseline, 3, 5, 7, 9, 11, 13, and 15 weeks. The arrow shows a significant decrease in SBP in the DH and ISO group when compared with DH group at 13 and 15 weeks. (**B**) The evaluation of the heart rate and systemic hemodynamics, with the evaluation of renal blood flow and renal vascular resistance at 120 days. Data are reported as mean ± standard error. The significance level for a null hypothesis was set at 5% (*p* < 0.05). (₸) compared to the CTL group at 120 days and (₸₸) compared to the DH group at 120 days.

The DH group and the DH and ISO group (Figure 2A) showed an increase in systolic blood pressure compared to that of the control group after week three of the study. Notably, the DH and ISO group presented a significant decrease in SBP when compared to the DH group after week 13.

Figure 2B also shows the heart rate and systemic hemodynamics of the groups in the final period of the experiment (120 days). We observed a decrease in RBF and an increase in RVR in the DH group. Interestingly, the rats exposed to the DH diet for 120 days and treated concomitantly with isoflavones in the last 60 days had RBF and RVR rates similar to those of the control group.

**Figure 3:**
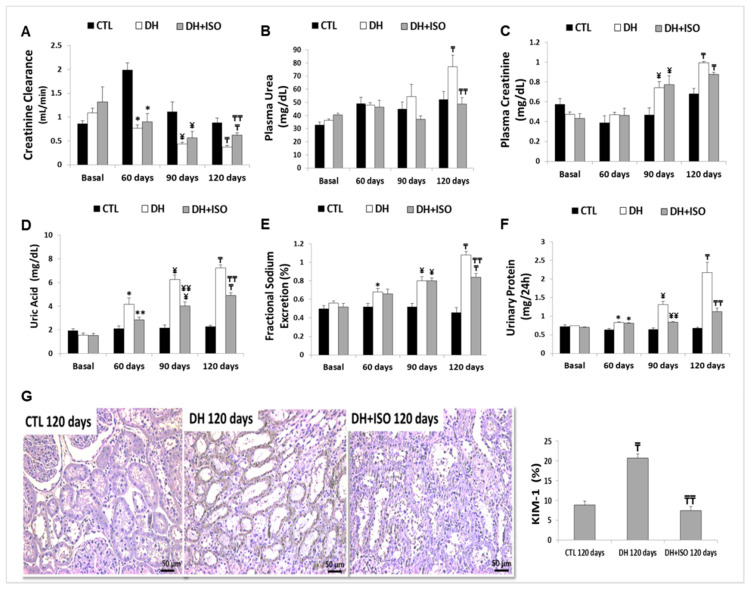
Evaluation of renal function. Rats in the CTL group, the DH group, and the DH and ISO group at baseline, 60, 90, and/or 120 days. (**A**) Creatinine clearance, (**B**) plasma urea, (**C**) plasma creatinine, (**D**) uric acid, (**E**) fractional sodium excretion, and (**F**) urinary protein. (**G**) Light microscopy of kidney injury molecule-1 (KIM-1) and quantitative analyses of kidney sections stained for KIM-1. Data are reported as mean ± standard error. The significance level for a null hypothesis was set at 5% (*p* < 0.05). (*) compared to the CTL group at 60 days, (**) compared to the DH group at 60 days, (¥) compared to the CTL group at 90 days, (¥¥) compared to the DH group at 90 days, (₸) compared to the CTL group at 120 days, and (₸₸) compared to the DH group at 120 days.

As shown in Figure 3A, we showed a significant decrease in creatinine clearance in all the experimental groups when compared to the CTL group at the same experimental period. When compared to the DH group, the DH and ISO group showed an increase in creatinine clearance at 120 days.

When compared to the CTL diet group at 120 days, the high-fat/high-fructose diet stimulated a significant increase in the plasma urea (Figure 3B). Interestingly, the plasma urea in the DH and ISO group was significantly decreased at 120 days compared to that in the DH group and was similar to those of the control group.

Both groups showed a significant increase in the plasma creatinine (Figure 3C) at 90 and 120 days when compared to the CTL group at the same experimental period. No statistical difference was found between the groups treated or not treated with isoflavones.

We observed increased levels of plasma uric acid (Figure 3D) in all experimental groups after 60 days of the study when compared to the CTL group at the same experimental period. Nevertheless, the rats exposed to the DH diet for 120 days and treated concomitantly with isoflavones for the last 60 days presented lower levels of uric acid on days 90 and 120 than the rats in the DH group for the same experimental period.

Tubular function was evaluated based on the levels of sodium excretion (Figure 3E). Increased sodium excretion was observed in the DH group after 60 days and in the DH and ISO group after 90 days when compared to the CTL group at the same experimental period. Again, the DH and ISO group showed an improvement, as there was a decrease in sodium excretion at 120 days when compared to that in rats on the DH diet for the same experimental period. 

We also observed a significant increase in urinary protein excretion (Figure 3F) in the rats fed the DH diet at all experimental periods, whereas the DH and ISO group showed an increase only before the treatment with isoflavones (60 days) in comparison to the CTL group at the same experimental period. 

The marker of renal injury (KIM-1) was also evaluated at 120 days [65]. As shown in Figure 3G, we observed an increase in KIM-1 in the rats on the DH diet when compared to that in the rats on the CTL diet. Notably, the DH and ISO group was not statistically different to the CTL group.

### 3.3. Analysis of the Kidney Cortex

**Figure 4:**
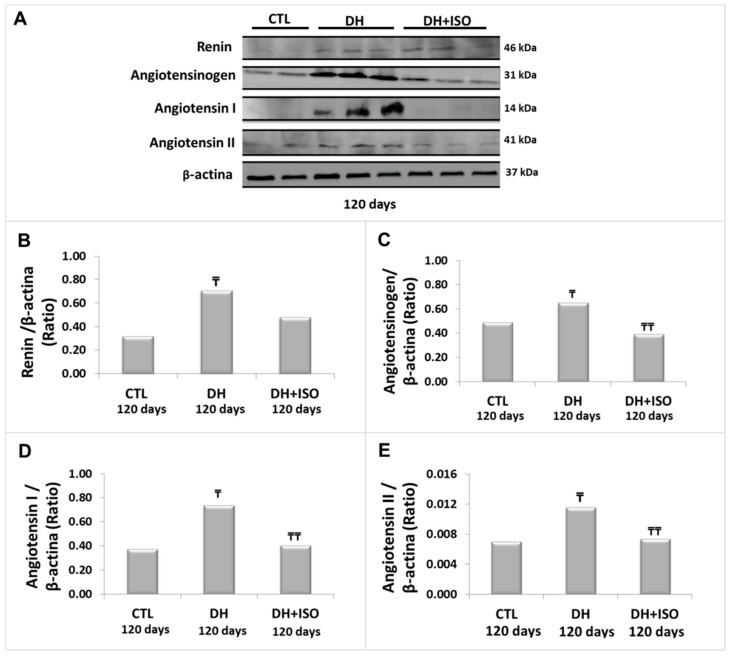
Renin–angiotensin system in the kidney cortex. Rats in the CTL group, the DH group, and the DH and ISO group at 120 days. Western blot images (**A**) for renin, angiotensinogen, angiotensin I, and angiotensin II. Quantitative analyses of immunoblotting images were performed using ImageJ for renin (**B**), angiotensinogen (**C**), angiotensin I (**D**), and angiotensin II (**E**). Data are reported as mean ± standard error. The significance level for a null hypothesis was set at 5% (*p* < 0.05). (₸) compared to the CTL group at 120 days and (₸₸) compared to the DH group at 120 days.

Figure 4 shows the immunoblot analysis with their respective graphical quantifications at 120 days of renin (Figure 4A,B), angiotensinogen (Figure 4A,C), angiotensin I (Figure 4A,D), and angiotensin II (Figure 4A,E). The group fed the DH diet for 120 days showed the activation of RAS. Inversely, the rats exposed to the DH diet for 120 days and treated concomitantly with isoflavones after 60 days (DH and ISO) did not exhibit statistically different effects when compared to the CTL group.

**Figure 5:**
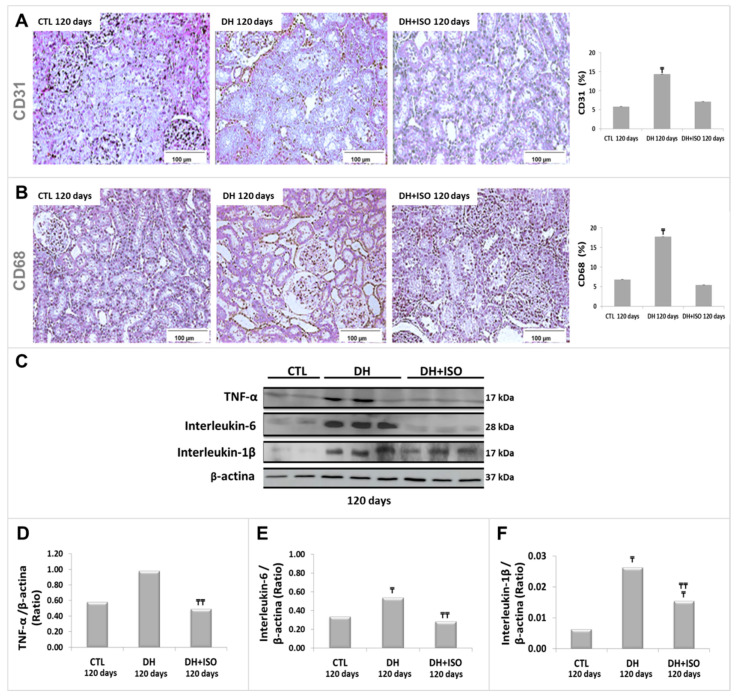
Inflammatory markers in the kidney cortex. Rats in the CTL group, the DH group, and the DH and ISO group at 120 days. Light microscopy images of the kidney sections immunostained for cluster of differentiation 31 (CD31) **(A**) and CD68 (**B**), as well as their quantitative analyses. Western blot images (**C**) for tumor necrosis factor (TNF-α), interleukin (IL-6), and interleukin-1β (IL-1β). Quantitative analyses of immunoblotting images were performed using ImageJ for TNF-α (**D**), IL-6 (**E**), and IL-1β (**F**). Data are reported as mean ± standard error. The significance level for a null hypothesis was set at 5% (*p* < 0.05). (₸) compared to the CTL group at 120 days and (₸₸) compared to the DH group at 120 days.

Figure 5 shows immunostaining results for CD31 (Figure 5A) and CD68 (Figure 5B), as well as immunoblot analyses with their respective graphical quantifications at 120 days for TNF-α (Figure 5C,D), IL-6 (Figure 5C,E), and IL-1β (Figure 5C,F) in the kidney tissue. The DH group showed an increase in the levels of all inflammation mediators compared to those in the CTL group. In contrast, the DH and ISO group did not show statistically different effects when compared to the CTL group, except for the expression of IL-1β, which increased in comparison to that in the CTL group but decreased in comparison to that in the DH group. 

**Figure 6:**
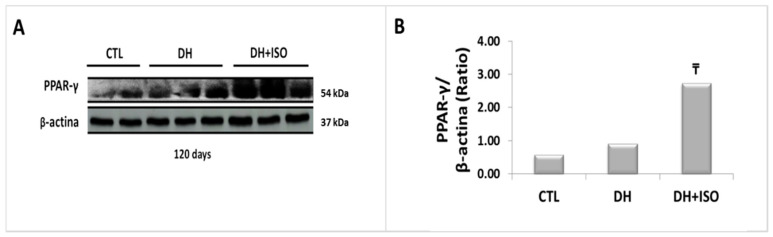
Peroxisome proliferator-activated receptor (PPAR-γ1) signaling in the kidney cortex. Rats in the CTL group, the DH group, and the DH and ISO group at 120 days. (**A**) Western blot images. (**B**) Quantitative analyses of the immunoblot images were performed using ImageJ. Data are reported as mean ± standard error. The significance level for a null hypothesis was set at 5% (*p* < 0.05). (₸) compared to the CTL group at 120 days and (₸₸) compared to the DH group at 120 days.

Figure 6 shows the immunoblot analyses of PPAR-γ (Figure 6A) with their respective graphical quantifications (Figure 6B) at 120 days. Only rats exposed to the DH diet for 120 days and treated concomitantly after 60 days with isoflavones (DH and ISO) showed the stimulated expression of PPAR-γ in the kidney.

### 3.4. Oxidative and Nitrosative Stress in Urine 

**Figure 7:**
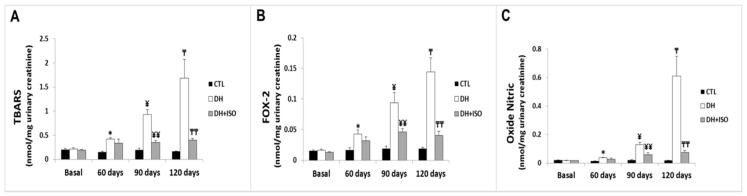
Analysis of the formation of reactive nitrogen species (RNS) and reactive oxygen species (ROS) in urine. Rats in the CTL group, the DH group, and the DH and ISO group at baseline, 60, 90, and 120 days. (**A**) Quantitative analyses of thiobarbituric reactive substances (TBARS), (**B**) urinary peroxides (FOX-2), and (**C**) nitric oxide (NO). Data are reported as mean ± standard error. The significance level for a null hypothesis was set at 5% (*p* < 0.05). (*) compared to the CTL group at 60 days, (**) compared to the DH group at 60 days, (¥) compared to the CTL group at 90 days, (¥¥) compared to the DH group at 90 days, (₸) compared to the CTL group at 120 days, and (₸₸) compared to the DH group at 120 days.

Figure 7 shows the urinary levels of thiobarbituric acid-reactive substances (TBARS), urinary peroxides (FOX-2), and nitric oxide (NO). There was an increase in lipid peroxidation, as shown by the levels of TBARS (A), FOX-2 (B), and NO (C) in the DH group when compared to those in the CTL group for the same experimental period. Interestingly, the DH and ISO group did not show statistically different effects when compared to the CTL group during all experimental periods.

## 4. Discussion

In this study, we observed an effect of the combination of fructose and high-fat diet on systemic metabolism and kidney function. After two months of ingestion, there was an expansion in visceral fat associated with long-term metabolic disorders, such as the maintenance of hyperglycemia, insulin resistance, polyuria, dyslipidemia (hypercholesterolemia and hypertriglyceridemia), and hypertension. 

We investigated morphological and functional kidney responses in rats exposed to the DH diet. Changes in intrarenal hemodynamics were observed with an increase in the RVR and a reduction in the RBF. Together with the decrease in creatinine clearance and increased proteinuria, urea, sodium excretion levels, as well as the expression of tubular injury marker KIM-1, a decline in the renal function was confirmed. These effects were mediated through reactive oxygen species and reactive nitrogen species production. 

In the kidney of animals on the DH diet, the infiltration of inflammatory cells with the elevated expression of IL-1β, TNF-α, and IL-6 was observed. Inflammation is common in patients with CKD, and it is inversely related to kidney function and positively associated with the magnitude of proteinuria excretion [66].

In this pathological scenario, intrarenal RAS activation was observed. Angiotensin II stimulates several pathophysiological mechanisms, including the generation of oxidative stress, was in renal hemodynamics, and the activation of the immune system in the kidney [67], which is consistent with our results.

Notably, under our experimental conditions, treatment with soy isoflavones was able to revert most of the renal alterations observed in the obese rats, and PPAR-γ-expression could be involved in the effects exerted by isoflavones. 

Initially, we noticed to the improvement in blood pressure and RBF, along with a decrease in RVR. In addition, there was an increase in creatinine clearance, whereas proteinuria, urea, and creatinine levels were not statistically different when compared to those in the control group. The improvement in tubular function was confirmed by the decrease in sodium excretion associated with the normalization of KIM-1 expression.

PPAR-γ modulates a broad range of physiopathological processes, including lipid metabolism, cellular differentiation, and insulin sensitization [21,39]. Though predominantly expressed in the adipose tissue, PPAR-γ expression is also found in different regions of the kidney; it is expressed in multiple types of renal cells, including proximal epithelial cells, podocytes, and glomerular mesangial cells, and it is dominantly expressed in the collecting system of the mammalian urinary tract, including collecting ducts and connective renal tubules [39,68,69]. 

Indeed, PPAR-γ ligand full synthetic agonists are the main target of thiazolidinedione drugs and have been shown to have renoprotective effects in both diabetic and non-diabetic patients [70]. However, some full synthetic agonists showed adverse effects that led to their withdrawal from the market or the restriction of their clinical use [71]. 

Clinically, congestive heart and failure renal sodium retention are probably the most important and troublesome adverse side effects. Troglitazone has been excluded from the market because it has been found to cause fatal liver dysfunction, whereas rosiglitazone can selectively enhance vascular permeability in the retina and adipose tissues; it has also been associated with bone fractures. At present, the most critical controversy regarding thiazolidinedione drugs is the possibility that pioglitazone may cause bladder cancer [72,73].

Thus, extensive research efforts have been undertaken recently to explore the potential of selective PPAR-γ natural modulators that can elicit reduced side effects due to partial PPAR-γ agonism [74,75,76]. Notably, PPAR-γ-partial-ligand natural modulators are often identified in plants that are common food sources, including soybeans, ginger, wine, palm oil, and several culinary herbs and spices [74]. 

Our group also identified that soybeans isoflavones have PPAR-γ1 modulating activity. However, it is the first time a study has demonstrated the therapeutic effects of isoflavones in modulating PPAR-γ in the kidney. This activation in the kidney is probably the key to explain the benefits of the use of isoflavones in subjects with CKD in several published clinical studies [23,26,27,28,29]. 

About 80% of CKD patients have a renin–angiotensin system (RAS) hyperactivity associated with the progression of renal damage [77]. The group fed a DH diet for 120 days and treated concomitantly with isoflavones after 60 days did not exhibit different levels of RAS when compared to the CTL group.

With an important influence on the kidney function, the adipose tissue secretes all components of the RAS [77]. In obesity, there is a systemic overactivation of this process, and the glomerulus manifests high physical stress and consequently affects the glomerular filtration rate, especially in podocytes. The loss of podocytes is a chain reaction that leads to the loss of the entire glomerulus [78,79,80]. 

In particular, angiotensin II participates in the pathogenesis of renal diseases through the regulation of two key processes: fibrosis and inflammation. These dysregulated responses are the catalysts and/or mediators in the progression of renal disease [81].

Molinas et al. [82] showed that angiotensin II inhibitor losartan has anti-inflammatory activity through the inhibition of the leucocyte infiltration and production of IL-1β, TNF-α, and IL-6; thus, it provides renoprotection. Similarly, our data showed a decrease in leukocyte infiltration and macrophages with a low production of proinflammatory cytokines IL-1β, TNF-α, and IL-6 in the kidneys of rats. Notably, the levels of angiotensin II in the kidneys of obese rats treated with isoflavones remained stable, similar to those from the control group; PPAR-γ may be involved in this stability.

Regarding PPAR-γ and inflammation, some studies [83,84,85] have observed that the loss of PPAR-γ results in a defective resolution of inflammation with chronic leukocyte recruitment; however, its activation promotes the cessation of neutrophil recruitment in the lung, spleen, and adipocytes, as well as in sepsis. Interestingly, we demonstrated the partial resolution of inflammation and PPAR-γ activation in the kidney.

In relation to PPAR-γ and angiotensin II, Benson et al. [80] and Schupp et al. [86] showed that telmisartan could bind to the ligand-binding domain of PPAR-γ at a different site than that used by thiazolidinedione drugs and act as an angiotensin II antagonist, exerting beneficial effects on the kidney by decreasing inflammation and proteinuria. We observed similar results in this study with the use of isoflavones (angiotensin II, proteinuria, and inflammation). 

The renoprotective effects of angiotensin II blockers are complex and multiple. The reduction in blood pressure by systemic vasodilation contributes to its beneficial effects in the treatment of CKD. Furthermore, renal vasodilation results in an increase in RBF, leading to improvement in ischemia and renal hypoxia. These are also effective in reducing urinary protein excretion through a reduction in intraglomerular pressure and the protection of glomerular endothelium and/or from podocyte injuries, in addition to blocking angiotensin II-induced tissue injuries and renal cells [87]. 

Furthermore, the intrarenal RAS mediates fatty acid-induced endoplasmic reticulum stress in the kidney [88,89,90,91]. Owing to the massive recruitment of inflammatory cells to the kidney, the uptake of oxygen in the area increases, thus leading to an increase in the production of ROS, which may interfere with renal tubule ion transport by altering renal blood pressure while causing oxidative injury to the proximal tubules that may also induce the expression of profibrotic molecules [88,89,90,91]. In the urine of treated rats, there was a decrease in lipid peroxidation, urinary peroxides, and reactive nitrogen species.

We believe that the effect of isoflavones in the activation of PPAR-γ provides renoprotection in our experimental protocol. Collectively, our results support the therapeutic effects of isoflavones on high-fat/high-fructose diet-induced renal damage, possibly via PPAR-γ antagonizing angiotensin II. 

The potential role of PPARs in mediating the biological actions of isoflavones is gaining appreciation, and we believe that our study provides a basis for further studies to improve our understanding of the associated molecular events.

## 5. Conclusions

In conclusion, the study findings showed that the beneficial effects of isoflavones in the renal function of obese rats could be mediated by expression of PPAR-γ, possibly via PPAR-γ antagonizing angiotensin II in the kidney.

## Figures and Tables

**Table 1:** Metabolic parameters.

Food Consumption (g)	Basal	30 Days	60 Days	90 Days	120 Days
CTL	-	14.11 ± 0.88	15.17 ± 0.31	15.15 ± 0.34	15.54 ± 0.25
DH	-	17.83 ± 0.67 +	19.05 ± 0.10 *	19.08 ± 0.21 ¥	19.03 ± 0.33 ₸
DH+ISO	-	19.01 ± 0.28 ++	18.74 ± 0.51 *	18.93 ± 0.43 ¥	19.24 ± 0.30 ₸
**Food Calorie (Kcal)**	**Basal**	**30 days**	**60 days**	**90 days**	**120 days**
CTL	-	55.04 ± 3.48	59.15 ± 1.22	59.07 ± 1.31	60.61 ± 0.98
DH	-	100.76 ± 3.78 +	107.65 ± 0.55 *	107.80 ± 1.16 ¥	107.50 ± 1.84 ₸
DH+ISO	-	107.43 ± 1.56 ++	105.88 ± 2.89 *	106.94 ± 2.41 ¥	108.71 ± 1.70 ₸
**Weight (g)**	**Basal**	**30 days**	**60 days**	**90 days**	**120 days**
**CTL**	184.40 ± 5.22	198.25 ± 3.37	312.55 ± 5.41	353.20 ± 12.05	402.10 ± 11.08
**DH**	183.20 ± 5.23	222.45 ± 4.44 +	371.50 ± 17.98 *	464.00 ± 14.49 ¥	553.25 ± 18.76 ₸
**DH+ISO**	175.00 ± 6.63	203.20 ± 2.61 ++	365.70 ± 11.11 *	450.10 ± 12.99 ¥	520.05 ± 19.60 ₸

Food consumption (g), food calorie (Kcal), and weight (g) in rats in the control group (CTL), high-fat/high-fructose diet group (DH), and high-fat/high-fructose diet and isoflavones group (DH+ISO) at baseline, 30, 60, 90, and 120 days. Data are reported as mean ± standard error. The significance level for a null hypothesis was set at 5% (*p* < 0.05). (+) compared to the CTL group at 30 days, (++) compared to the DH group at 30 days, (*) compared to the CTL group at 60 days, (**) compared to the DH group at 60 days, (¥) compared to the CTL group at 90 days, (¥¥) compared to the DH group at 90 days, (₸) compared to the CTL group at 120 days, and (₸₸) compared to the DH group at 120 days.

**Table 2:** Lipid metabolism.

Visceral Fat (g)	Basal	60 Days	90 Days	120 Days
CTL	-	-	-	53.12 ± 5.50
DH	-	-	-	244.81 ± 11.78 ₸
DH+ISO	-	-	-	123.44 ± 5.75 ₸ ₸₸
**Triglycerides** **(mg/dL)**	**Basal**	**60 days**	**90 days**	**120 days**
CTL	130.00 ± 5.59	134.20 ± 3.12	135.60 ± 4.35	155.00 ± 3.96
DH	141.40 ± 4.79	163.20 ± 1.56 *	183.80 ± 2.03 ¥	189.80 ± 1.98 ₸
DH+ISO	122.80 ± 3,26	152.60 ± 4.30 *	165.20 ± 4.34 ¥ ¥¥	174.40 ± 3.14 ₸ ₸₸
**Total cholesterol** **(mg/dL)**	**Basal**	**60 days**	**90 days**	**120 days**
CTL	104.40 ± 5.76	107.00 ± 4.06	131.00 ± 4.56	133.40 ± 2.87
DH	94.20 ± 4.12	228.20 ± 6.86 *	260.40 ± 13.63 ¥	290.40 ± 4.21 ₸
DH+ISO	102.60 ± 8.58	170.80 ± 31.66 *	232.20 ± 20.92 ¥	165.60 ± 2.96 ₸ ₸₸
**HDL cholesterol** **(mg/dL)**	**Basal**	**60 days**	**90 days**	**120 days**
CTL	285.79 ± 2.12	250.14 ± 21.65	232.92 ± 26.24	245.80 ± 13.60
DH	236.94 ± 10.97	177.80 ± 10.14 *	140.80 ± 14.03 ¥	108.15 ± 1.66 ₸
DH+ISO	268.72 ± 3.99	209.80 ± 23.12	185,80 ± 16.13	187.22 ± 18.34 ₸ ₸₸

Visceral fat (g), triglycerides (mg/dL), total cholesterol (mg/dL), and HDL cholesterol (mg/dL) in rats in the control group (CTL), high-fat/high-fructose diet group (DH), and high-fat/high-fructose diet and isoflavones group (DH+ISO) at baseline, 60, 90, and 120 days. Data are reported as mean ± standard error. The significance level for a null hypothesis was set at 5% (*p* < 0.05). (*) compared to the CTL group at 60 days, (**) compared to the DH group at 60 days, (¥) compared to the CTL group at 90 days, (¥¥) compared to the DH group at 90 days, (₸) compared to the CTL group at 120 days, and (₸₸) compared to the DH group at 120 days.

**Table 3:** Glucose metabolism.

Glycated Hemoglobin (%)	Basal	30 Days	60 Days	90 Days	120 Days
CTL 120 days	-	-	-	-	4.48 ± 0.24
DH 120 days	-	-	-	-	8.12 ± 0.39 ₸
DH+ISO 120 days	-	-	-	-	6.50 ± 0.39 ₸ ₸₸
**Glucose** **(mg/mL)**	**Basal**	**30 days**	**60 days**	**90 days**	**120 days**
CTL	89.00 ± 5.70	91.0 7 ± 2.26	90.50 ± 2.55	92.00 ± 2.53	96.40 ± 2.27
DH	85.00 ± 2.28	112.27 ± 2.05 +	124.20 ± 2.75 *	124.30 ± 0.47 ¥	127.30 ± 1.90 ₸
DH+ISO	87.20 ± 5.34	116.80 ± 1.93 +	122.50 ± 1.76 *	118.30 ± 2.49 ¥	117.25 ± 0.93 ₸ ₸₸
**Polyuria** **(mL)**	**Basal**	**30 days**	**60 days**	**90 days**	**120 days**
CTL	9.20 ± 0.37	-	11.00 ± 0.95	9.60 ± 0.93	9.80 ± 0.86
DH	9.80 ± 0.37	-	11.40 ± 0.98	15.20 ± 0.37 ¥	18.20 ± 0.84 ₸
DH+ISO	9.40 ± 1.21	-	11.20 ± 0.92	10.80 ± 1.50 ¥	13.60 ± 1.03 ₸ ₸₸
**kITT** **(% min)**	**0**	**10 min**	**20 min**	**30 min**	**-**
CTL 120 days	99.25 ± 1.12	85.25 ± 2.49	75.50 ± 1.13	52.00 ± 1.41	-
DH 120 days	122.20 ± 1.85	108.40 ± 3.26 +	96.60 ± 5.41 *	78.60 ± 7.03 ¥	-
DH+ISO 120 days	108.00 ± 2.66	92.20 ± 3.02	81.80 ± 1.32	68.60 ± 4.68	-

Glycated hemoglobin (%), glucose (mg/mL), polyuria, and plasma glucose disappearance rate (kITT; (% min)) in rats in the control group (CTL), high-fat/high-fructose diet group (DH), and high-fat/high-fructose diet and isoflavones group (DH+ISO) at baseline, 30, 60, 90, and 120 days. Data are reported as mean ± standard error. The significance level for a null hypothesis was set at 5% (*p* < 0.05). (+) compared to the CTL group at 30 days or 10 min, (*) compared to the CTL group at 60 days or 20 min, (**) compared to the DH group at 60 days, (¥) compared to the CTL group at 90 days or 30 min, (¥¥) compared to the DH group at 90 days, (₸) compared to the CTL group at 120 days, and (₸₸) compared to the DH group at 120 days.
